# Case Report: *Campylobacter fetus* caused pyogenic spondylodiscitis with a presentation of cauda equina syndrome after instrumented lumbar fusion surgery

**DOI:** 10.3389/fsurg.2022.998011

**Published:** 2022-10-04

**Authors:** Matevž Topolovec, Nataša Faganeli, Peter Brumat

**Affiliations:** ^1^ Department of Spine Surgery, Valdoltra Orthopaedic Hospital, Ankaran, Slovenia; ^2^ Faculty of Medicine, University of Maribor, Maribor, Slovenia; ^3^ Faculty of Medicine, University of Ljubljana, Ljubljana, Slovenia

**Keywords:** *Campylobacter fetus*, spondylodiscitis, cauda equina syndrome, CES, instrumented lumbar fusion, decompression

## Abstract

Spondylodiscitis with/without neurologic impairment is a serious infection, predominantly occurring in high-risk patients. *Campylobacter fetus* caused spondylodiscitis is very rare. Evidence-based therapeutic concepts for lumbar spondylodiscitis are lacking. A 64-year-old high-risk woman underwent decompression with instrumented lumbar fusion. Six months after index surgery, she developed pyelonephritis, which deteriorated to sepsis and presentation of cauda equina syndrome. She underwent urgent revision with decompression, debridement, and instrumentation removal, and received long-term antibiotics. Culture grew *Campylobacter fetus*, previously not reported as a cause of spondylodiscitis after elective instrumented lumbar fusion. Emergent debridement and removal of instrumentation, with 2 months of targeted intravenous antibiotics followed by 6 weeks of oral antibiotics led to complete spondylodiscitis resolution. Prompt diagnostics and targeted antibiotic treatment are paramount when dealing with spinal infections, particularly in patients with rare causative pathogens like *Campylobacter fetus*. Concomitant neurological complications may require emergent surgical management in the case of cauda equina syndrome.

## Introduction

Infections after instrumented spinal surgeries represent a major morbidity cause, incur substantial costs to the healthcare system, and may be associated with serious long-term sequelae (1). Although spondylodiscitis is a well-reported serious infection whereby high-risk patients are predominantly affected, *Campylobacter fetus* (CF) caused spondylodiscitis is very rare (1–9). Concomitant neurologic impairment may lead to severe consequences if the diagnosis is delayed (7). Cauda equina syndrome (CES) is a potentially devastating spinal condition, in which emergent spinal surgery referral is indicated (10). We report a rare case of CF-caused pyogenic spondylodiscitis with a presentation of CES after instrumented lumbar fusion surgery.

## Case description

**Figure 1:**
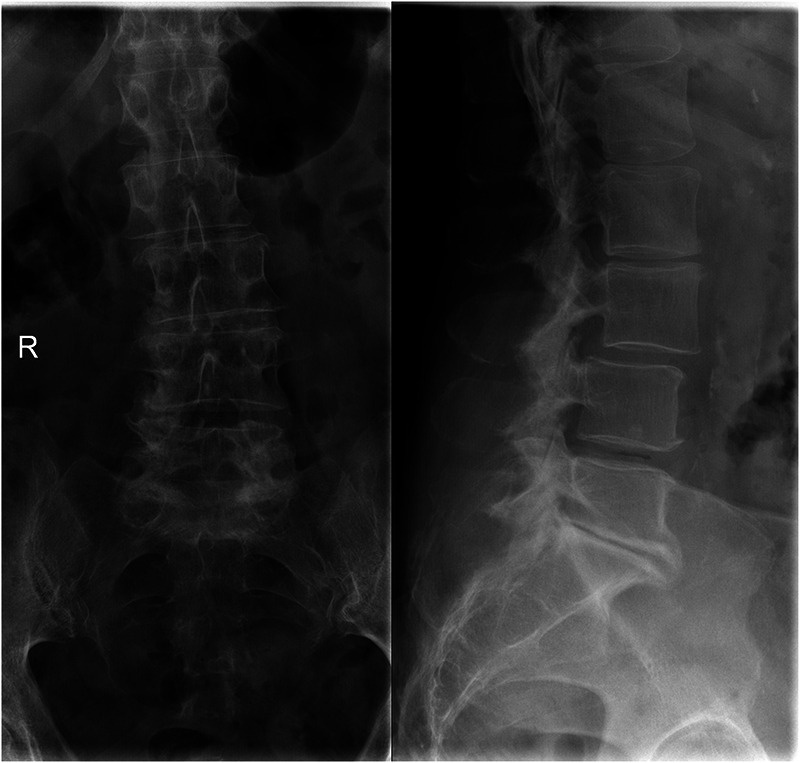
Preoperative standing x-ray prior to index surgery: anteroposterior view (left) and lateral view (right).

**Figure 2:**
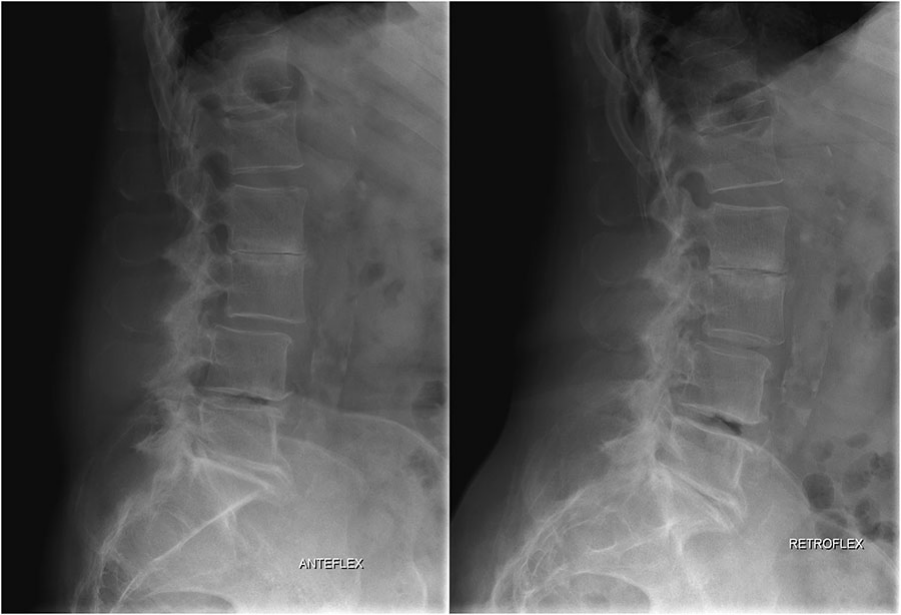
Evaluation of lumbar spine motion with dynamic x-ray prior to index surgery: flexion view (left) and extension view (right).

**Figure 3:**
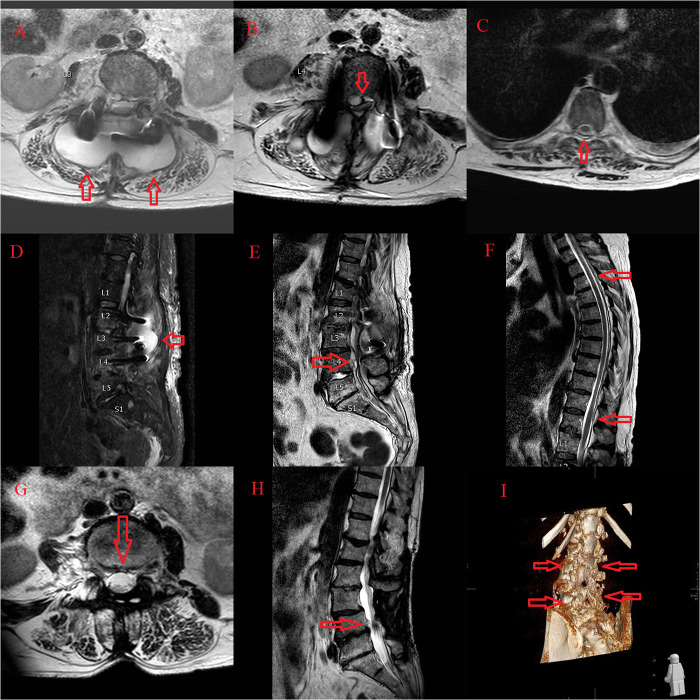
Emergent MRI demonstrated pyogenic spondylodiscitis with paraspinal and epidural purulent collection at the level of spondylodesis L2 to L4 (**A,D**) with total spinal stenosis at the L4 level (**B,E**). Concomitant purulent collections, without neurologic compromise, from the Th4 to Th12 level (**C,F**). The follow-up imaging revealed residual fluid collections in the paraspinal soft tissue and right psoas muscle, with regression of spondylodiscitis, resolution of total spinal stenosis at the L4 level (**G,H**), and evidence of bone fusion with postoperative deformity at the level of surgery (**I**).

A 64-year-old woman with history a of chronic obstructive pulmonary disease, asthma, elevated arterial blood pressure, eczematous dermatitis, and alcohol abuse was operated on at our institution for decompression with posterolateral L2–L4 instrumented fusion, due to stenosis and spondylolisthesis of L3 (Figures 1, 2). The postoperative course was unremarkable. Six months after the index surgery she was referred back to our institution due to low back pain, leg weakness, and impaired bowel and bladder function, from an outside facility where she was treated for *Escherichia coli* caused pyelonephritis with the intravenous antibiotic cefepime 1 g/12 h, followed by intravenous imipenem/cilastatin 500 mg/500 mg/6 h due to later CF bacteremia. There was no clinical or trans-esophageal echocardiographic evidence of endocarditis at presentation. Her serum C-reactive protein was 137 mg/L. Emergent MRI demonstrated pyogenic spondylodiscitis with paraspinal and epidural purulent collection at the level of spondylodesis L2–L4 with total spinal stenosis at the L4 level (Figure 3). Concomitant purulent collections, without neurologic compromise, were seen at the Th4–Th12 levels and in the right psoas muscle (Figure 3). After stabilizing the patient due to sepsis, we performed an urgent decompression surgery with the removal of all spinal implants. Intraoperative exploration revealed complete loosening of the titanium pedicle screws with formed but delicate posterolateral bone fusion. Tissue biopsy confirmed CF spondylodiscitis by broad-range polymerase chain reaction (BR-PCR) test. Following the surgery, the bowel function, low back pain, and leg weakness slowly recovered, but the bladder dysfunction, unfortunately, remained unchanged. The patient was mobilized with a walker with the assistance of a physical therapist and remained wheelchair-bound for longer distances. Empirical ultrasound of the abdomen showed a structurally altered liver, otherwise without obvious pathomorphological changes. The follow-up imaging revealed residual fluid collections in the paraspinal soft tissue and right psoas muscle, with regression of spondylodiscitis, resolution of total spinal stenosis at the L4 level, and evidence of bone fusion with postoperative deformity at the level of surgery (Figure 3). After 2 months of intravenous therapy with imipenem/cilastatin, the patient was discharged with oral therapy with ciprofloxacin 500 mg/12 h for another 6 weeks and with an improved neurological condition and function.

**Figure 4:**
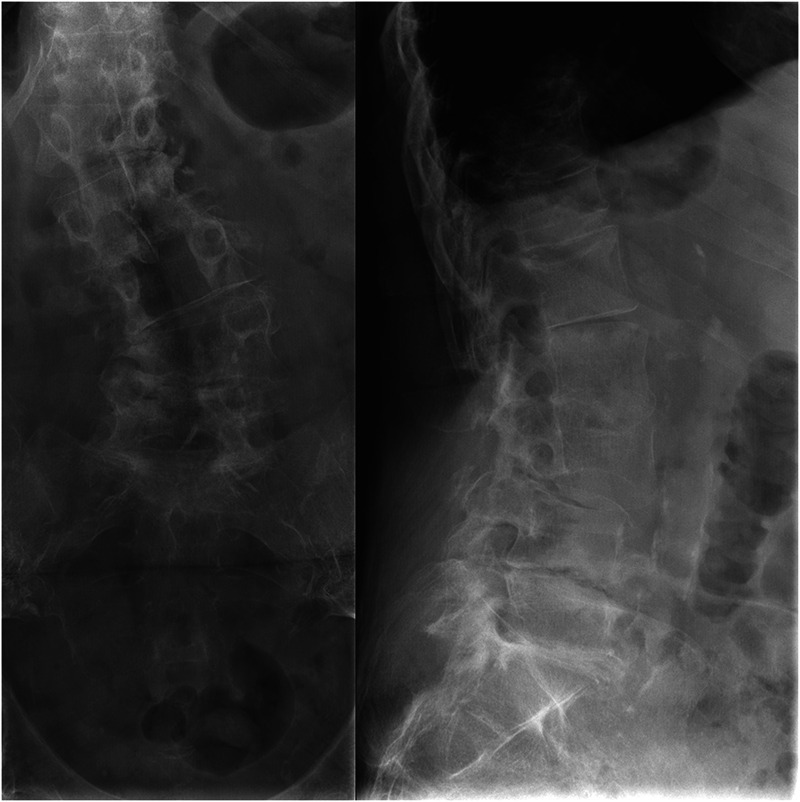
At the last follow-up, 4 years after the second surgery, the patient was still complaining of low back pain but was manageable with conservative methods, despite radiological progression of deformity: anteroposterior view (left) and lateral view (right).

At 1 year of follow-up, laboratory findings were within the normal range, she reported minor low back pain with improved bowel, bladder function, and ambulation. MRI showed no changes compared to the previous imaging. PET/CT and scintigraphy with marked leukocytes were both negative for an infection. At the last follow-up, 4 years after the second surgery, the patient was still complaining of low back pain but was manageable with conservative methods, despite radiological progression of deformity (Figure 4). No evident clinical signs of infection were seen.

## Discussion

Spondylodiscitis may represent a diagnostic challenge, especially with clinical onset without any specific signs and symptoms, of subacute or chronic presentation, and require thoughtful evaluation of radiological, biochemical, and microbiological examinations (1, 7). MRI remains the gold standard to confirm the diagnosis (1, 5). Evidence-based therapeutic concepts for infectious spondylodiscitis of the lumbar spine are still lacking (11). However, the latest publications discuss the rationale for using newly developed classification systems in improving the treatment of spondylodiscitis (12, 13).

CF-caused pyogenic spondylodiscitis is very rare, whereby reported cases predominantly occurred in high-risk patients, as did in our reported case, but contrary to our case, without previous instrumented lumbar fusion surgery (1–8). The patient in our case also had no obvious contact with infected animals or their products, which is a usual mode of transmission in such infection (3, 4, 6, 8).

The optimal treatment has not yet been defined for CF infective spondylodiscitis, although erythromycin and fluoroquinolones are the recommended treatment for systemic campylobacteriosis (5, 14). Nevertheless, it is paramount to accurately identify the causative pathogen in order to effectively prescribe targeted antimicrobial therapy and to select antibiotics that have good tissue penetration for such infections (5). In our case, we only had BR-PCR test confirmation, without any susceptibility data. We treated the patient with intravenous imipenem/cilastatin 500 mg/500 mg every 6 h, given its strong bactericidal effect and successful use in other reported CF infections (15). Furthermore, reported bone concentrations of the drug likely exceed target minimum inhibitory concentrations with higher bone penetration in case of infected that uninfected bone (16–18). After 2 months, we switched to oral therapy with ciprofloxacin 500 mg/12 h for another 6 weeks. Showing good penetration profiles, both carbapenems and fluoroquinolones have the ability to penetrate into rigid bone tissues, as well as the synovial fluid (18).

Additionally, percutaneous abscess drainage as an adjunct to traditional treatment may drastically reduce the focus of infection without the requirement for additional antibiotics (5). This was not considered in our case, because our patient needed urgent decompression of total stenosis and CES. Once suspected, emergent spinal surgery referral is indicated, along with urgent decompression, but even with expeditious surgery, improvements remain inconsistent (10). The understanding of the severity of CES is paramount because early intervention has been shown to portend a greater chance of neurologic recovery (10). This implies that a delay in diagnosis of lumbar spondylodiscitis and initiation of treatment may result in an intraspinal extension of the infection, with the resultant potential for devastating neurological complications, as were encountered in our case.

Prior to removing all of the spinal instrumentation, we intraoperatively confirmed solid, although delicate, bone fusion. However, in our case, additional fusion would make sense given the postoperative onset of deformity. Management of spontaneous thoracic and lumbar spondylodiscitis by surgical debridement and posterolateral open transpedicular fixation seems to be an effective and safe method despite the presence of infection (19). Patients with one or more relevant chronic concomitant diseases showed faster recovery, shorter hospital stays, and earlier return to daily routine after early dorsal fusion than after late dorsal fusion or abscess evacuation alone (11). Although the conventional open approach is the usual standard of care, employing percutaneous pedicle screw fixation to treat pyogenic spondylodiscitis of the thoracic and lumbar spine may be associated with significantly reduced operating time, blood loss, postoperative pain, length of stay, and rates of wound infection than open pedicle screw fixation (20). A retrospective cohort study comparing the safety and efficacy of minimally invasive and open surgery over a 9-year period by Viezens et al. (21) suggests that minimally invasive surgery is safe and effective for the treatment of spontaneous pyogenic thoracic and lumbar spondylodiscitis as well.

## Conclusions

Prompt diagnostics and targeted antibiotic treatment are paramount when dealing with spinal infections, particularly in patients with rare causative pathogens like CF. Neurological impairment may require emergent surgical management in the case of CES.

## Data Availability

The original contributions presented in the study are included in the article/Supplementary Material, further inquiries can be directed to the corresponding author.
